# Combined Flow Control Strategy Investigation for Corner Separation and Mid-Span Boundary Layer Separation in a High-Turning Compressor Cascade

**DOI:** 10.3390/e24050570

**Published:** 2022-04-19

**Authors:** Hejian Wang, Bo Liu, Xiaochen Mao, Botao Zhang, Zonghao Yang

**Affiliations:** 1 School of Power and Energy, Northwestern Polytechnical University, Xi’an 710129, China; hejian_wang@126.com (H.W.); liubo704@nwpu.edu.cn (B.L.); zhangbo_tao@126.com (B.Z.); yangzonghao@mail.nwpu.edu.cn (Z.Y.); 2 The National Key Laboratory of Science and Technology on Aerodynamic Design and Research, Xi’an 710129, China

**Keywords:** corner separation, mid-span boundary layer separation, blade-end and whole-span slots, end-wall boundary layer suction, static pressure rise coefficient, total pressure loss

## Abstract

To comprehensively control the corner separation and mid-span boundary layer (BL) separation, this study proposed and evaluated two new flow control configurations. One is a slotted configuration composed of blade-end and whole-span slots, and the other is a combined configuration with end-wall BL suction and whole-span slot. Additionally, the adaptability of the combined configuration to the lower blade solidity (c/t) condition was verified. The results indicate that both the slotted configuration and combined configuration can eliminate the mid-span BL separation, but a better reduction in the corner separation can be observed in the combined configuration. The two configurations can remove the concentrated shedding vortex and reduce the passage vortex (PV) for the datum cascade, but the wall vortex (WV) will be generated. By contrast, the combined configuration has weaker WV and PV than the slotted configuration, which contributes to further reducing the corner separation. In the combined configuration with a c/t of 1.66 and 1.36, the total pressure loss is reduced by 38.4% and 42.1%, respectively, on average, while the averaged static pressure rise coefficient is increased by 16.2% and 17.6%, respectively. This is advantageous for enhancing the working stability and pressure diffusion capacity for compressors. Besides this, the combined configuration with lower c/t can achieve a stronger pressure diffusion capacity and smaller loss than the higher c/t datum cascade. Therefore, the combined configuration is advantageous to the improvement of the aero-engine thrust-to-weight ratio through decreasing the compressor single-stage blade number.

## 1. Introduction

As two main flow separation phenomena in compressor stator blade passages, three-dimensional (3D) corner separation [1,2] and mid-span boundary layer (BL) separation [3] have always restricted the improvement of compressor performance, especially 3D corner separation. Severe corner separation will cause massive total pressure loss and serious passage blockage [4,5]. Therefore, understanding the flow mechanism of corner separation is extremely important and many relevant studies have been performed. Zambonini et al. [6,7] investigated the corner separation dynamics through experiments based on a compressor stator cascade. Liu et al. [8] studied the turbulence characteristics and vortical structures of corner separation by delayed detached-eddy simulations.

To improve the total pressure ratio, efficiency and stability margin for compressors, effectively controlling the 3D corner separation and mid-span BL separation is necessary. In recent years, some internal flow control methods have shown a good effect for the controlling of corner separation and mid-span BL separation [9]. Up to now, the main internal flow control methods have consisted of two branches (active and passive control methods). The former includes mainly BL suction [10,11], blowing [12,13], plasma excitation [14,15], etc., while the latter includes mainly blade slotting [16,17], end-bend blades [18,19], casing treatment [20,21], vortex generators [22,23], etc.

The flow near the blade pressure side can be introduced into a suitable slot configuration and accelerated to form a high-momentum jet at the blade suction side, thus effectively suppressing local flow separation by the re-energization of the low-momentum fluid. Hence, blade slotting has been widely applied by many researchers to control flow separation. Rockenbach et al. [24] proposed the blade slotting concept at first and conducted related experiments in a single-stage compressor; the results indicated that the total pressure ratio and efficiency were improved since the slot jet well suppressed the flow separation in the compressor.

Additionally, Ramzi et al. [25] simulated different slot geometries and studied their impact on the cascade performance. In the cascade with a suitable slot configuration, the maximum increment of flow turning angle was 5° while the total pressure loss could be maximumly reduced by 28%. Based on a compressor cascade with high loading, the effect of a blade-end slot was investigated by Liu et al. [26]. It was found that the 3D corner separation could be significantly controlled by the blade-end slot jet, thus leading to a great reduction in the total pressure loss. Tang et al. [27] applied four blade-end slots in a high-load compressor cascade. Under the action of the blade-end slot jet, the corner stall was delayed and the total pressure loss was greatly reduced.

Although much research on blade slotting has been carried out, most of it only controlled the corner separation through the high-momentum slot jet and did not consider the controlling of the mid-span BL separation. The research of Wang et al. [28] indicated that for a large-camber compressor blade with both corner separation and mid-span BL separation, controlling the corner separation by blade-end slotting will worsen the flow fields near the blade mid-span. As a result, it is necessary to control the corner separation while guaranteeing control for the mid-span BL separation.

However, the flow separation can be more effectively eliminated by the slot jet when the distance between the slot outlet and the separation point is relatively small [29,30]. Generally, the mid-span BL separation point is closer to the blade trailing edge (TE) than the corner separation point. To guarantee the slot jet behaves well in controlling the mid-span BL separation, the slot outlet should not be set too close to the blade leading edge (LE). Under such circumstances, the end-wall (EW) secondary flow will cause a certain secondary flow loss by developing and separating before the slot outlet [28,30]. To reduce the secondary flow loss to the greatest extent, this study proposed two new flow control strategies: slotted and combined configurations.

Besides this, as the ratio of blade chord (c) to pitch (t), blade solidity (c/t) was identified as an important parameter in compressor design [31,32]. Keeping c constant, generally, the decrease in c/t will lead to higher blade loading and larger separation. However, lower c/t contributes to a lower single-stage blade number. It means that if the compressor pressure diffusion capacity could be kept unchanged through some flow control methods, the decrease in c/t would help to improving the thrust-to-weight ratio for aero-engines. Therefore, it is worthwhile to verify the newly proposed flow control strategy at a condition with a lower c/t.

This paper is organized as follows: (1) Firstly, the geometric designs of the two new flow control configurations, the computational methods and validations are briefly introduced in Section 2. (2) Next, the new flow control mechanisms and effects are revealed and evaluated in detail in Section 3. (3) Subsequently, the adaptability of the combined configuration to the lower c/t condition is verified in Section 4. (4) Finally, the overall work is concluded in Section 5.

## 2. Configurations and Computational Method

### 2.1. Datum Cascade Configuration

**Figure 1:**
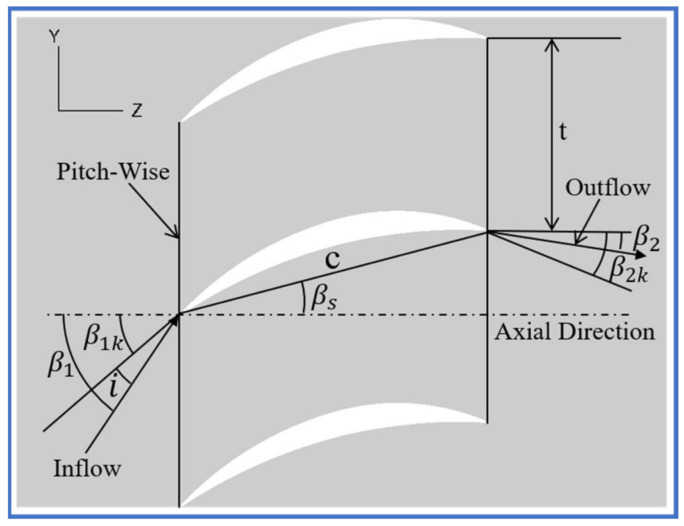
Two-dimensional blade profile of the datum cascade.

To better investigate the new proposed flow control strategies, a high-turning compressor cascade was selected by this study as the datum cascade. Figure 1 shows the two-dimensional blade profile for the datum cascade; its camber angle is as large as 53.38°. The datum cascade exhibits severe corner separation, while BL separation also exists around the datum cascade midspan because of the large camber angle. Therefore, the datum cascade is well suited for evaluating the newly proposed flow control strategies. Table 1 lists the detailed geometric and aerodynamic parameters for the datum cascade. One can see that the inlet Mach number was selected as 0.7 during simulations, and the Reynolds number ($Re_{C}$) according to the blade chord was $7.7\times 10^{5}$.

### 2.2. Flow Control Strategies

**Figure 2:**
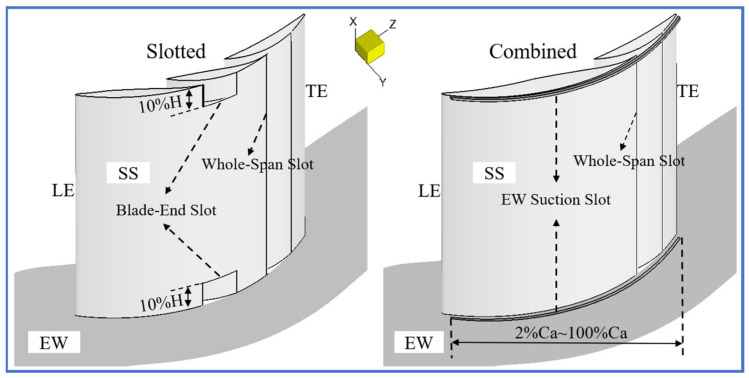
Slotted and combined flow control configurations.

Figure 2 shows the geometric configurations of the two new flow control strategies proposed in this study. According to previous studies [29,30], the smaller the distance between the slot outlet and the separation point, the more effective control effect on flow separation that can be achieved. Therefore, in order to effectively remove the midspan BL separation while reducing the corner separation to some extent, this study located the whole-span slot outlet at 75% of the axial blade chord (Ca) in both the slotted and combined configurations. To restrict the EW secondary flow migration toward the mid-span before the whole-span slot, this study applied two blade-end slots prior to the whole-span slot in the slotted configuration, and the blade-end slot occupied 10% of the blade height (H).

Additionally, the superiority of the EW BL suction in controlling the EW secondary flow development has been widely proved [33,34], and the EW BL suction shows a better engineering feasibility than the blade suction surface (SS) BL suction. Consequently, this study combined two EW suction slots with one whole-span slot in the combined configuration. To control the EW secondary flow development in essence over the whole chord length, the EW suction slot was set as 2%Ca to 100%Ca. Besides this, refer to the EW suction slot configuration in [34]; the EW suction slot owned the width of 1 mm and the distance between the EW suction slot and the blade SS was also 1 mm.

**Figure 3:**
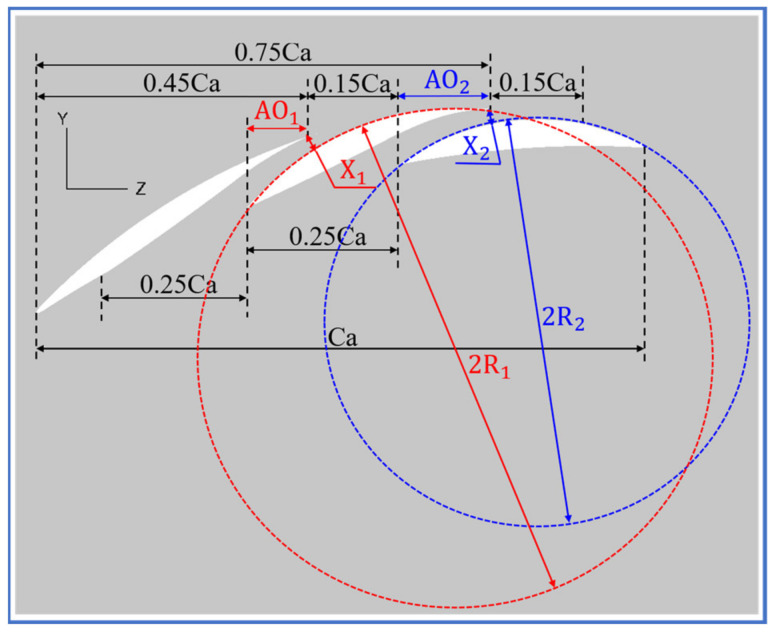
Two-dimensional blade profile at 5%H of the slotted configuration.

To better illustrate the whole-span and blade-end slot configurations, Figure 3 shows the two-dimensional blade profile cut from 5%H of the slotted configuration. It can be found that the whole-span slot and blade-end slot, respectively, had inlet and outlet widths of 0.25Ca and 0.15Ca. The outlet position of the blade-end slot was set as 45%Ca, which is relatively near the corner separation line (SL) starting point, thus causing the slot jet to behave well in controlling the corner separation.

In addition, to introduce the main stream near the blade pressure surface (PS) to the slot with less loss, the upper walls of the blade-end and whole-span slots were tangent with the blade PS, in accordance with [35]. Denton’s study [36] showed that the slot jet and the main flow will mix and bring a certain loss, and the corresponding mixing loss becomes larger as the slot jet angle increases. Therefore, the lower walls of the blade-end and whole-span slots were tangent with the blade SS, ensuring that the slot jet angle was basically the same with the main flow direction.

$X_{1}$ and $X_{2}$, respectively, represent the outlet throat widths for the whole-span and blade-end slots, while $R_{1}$ and $R_{2}$, respectively, represent their lower wall outlet curve radiuses. To guarantee the slot jet does not separate at the slot outlet curved surface, the ratios of $X_{1}$/$R_{1}$ and $X_{2}$/$R_{2}$ were both set as a small value (0.04) [37]. In addition, this setting further guarantees the identity of the slot jet angle with the mainstream direction, too. For the same purpose, the axial overlap ${\mathrm{AO}}_{1}$ of the blade-end slot and the axial overlap ${\mathrm{AO}}_{2}$ of the whole-span slot were set as large as 10%Ca and 15%Ca, respectively.

### 2.3. Computational Details

In this study, the numerical software of Fine/Turbo [38] was adopted to conduct the 3D Reynolds-Averaged Navier–Stokes (RANS) numerical simulations. In the computations, the explicit time marching four-step Runge–Kutta procedure solved the RANS equations in a single cascade passage while the periodicity boundary condition was applied. The space discretization was solved by a second-order cell-centered explicit finite volume scheme, which had four-order artificial dissipation. Many convergence acceleration methods including the implicit smoothing method, full multigrid algorithm, local time stepping method, etc., were applied.

In the previous studies [34,39], the flow fields of the datum cascade could be well captured by the Spalart–Allmaras (SA) turbulence model [40], hence the SA turbulence model was used in the present simulations. Additionally, a uniform inlet flow condition with an absolute total pressure (101,325 Pa) and total temperature (288.15 K) was set at the computational domain inlet boundary, and −6°~7° was selected as the inlet incidence angle range. The inlet Mach number of 0.7 was ensured by adjusting the static pressure at the computational domain outlet boundary. The simulations were judged to be convergent when the global mean residuals were less than $1\times 10^{-6}$.

**Figure 4:**
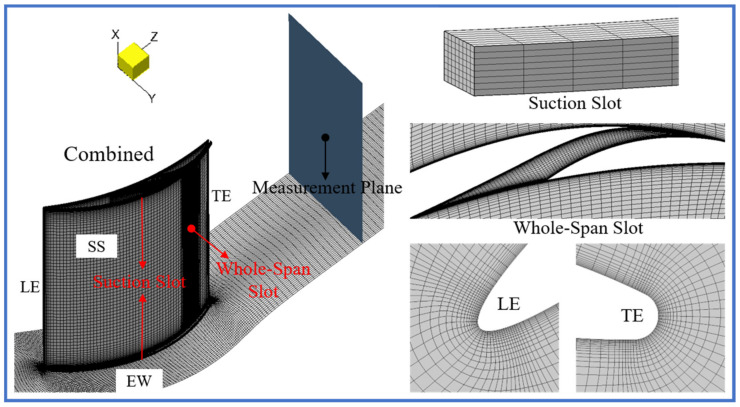
Overall and partial meshes of the combined configuration.

Figure 4 shows the overall and partial meshes of the combined configuration. The meshes in the mainstream passage of the datum cascade were automatically generated by AutoGrid5 [41], while the “H” type structured meshes of the whole-span slot, blade-end slot and EW BL suction slot were manually generated by IGG [42]. In the computational domain, the inlet and outlet boundaries were located 1.0C upstream and 3.0C downstream, respectively, from the blade LE. Additionally, this study selected the plane situated 2.0C downstream from the blade LE as the outlet measurement plane, and the corresponding cascade performance parameters were used for analysis and evaluation.

For obtaining a high mesh quality, the meshes of the datum cascade were modeled by “O4H-type” topologies and included 3,000,000 grid points. Besides this, a non-slip adiabatic wall condition was applied to both the blade and EW. To better simulate the BL, this study refined the meshes on the blade surface and the meshes near the EW with a minimum grid spacing value ($5\times 10^{-3}$ mm), ensuring that the dimensionless wall first layer grid size ($y^{+}$) was basically less than 1.

The meshes in the whole-span and blade-end slots included 1,030,000 and 320,000 grid points, respectively, and the non-slip adiabatic wall condition was applied to the slot upper and lower walls. The slot upper wall surface meshes and lower wall surface meshes were refined for better capturing the slot BL, and a full non-matching connection technology was applied to connect the meshes in the slot and the meshes on the blade surface. In addition, this study refined the meshes around the slot inlet and outlet, ensuring a high quality of numerical value transfer in simulations. Meanwhile, for capturing the BL in the slot near the EW, this study also refined the slot meshes along the blade span.

**Figure 5:**
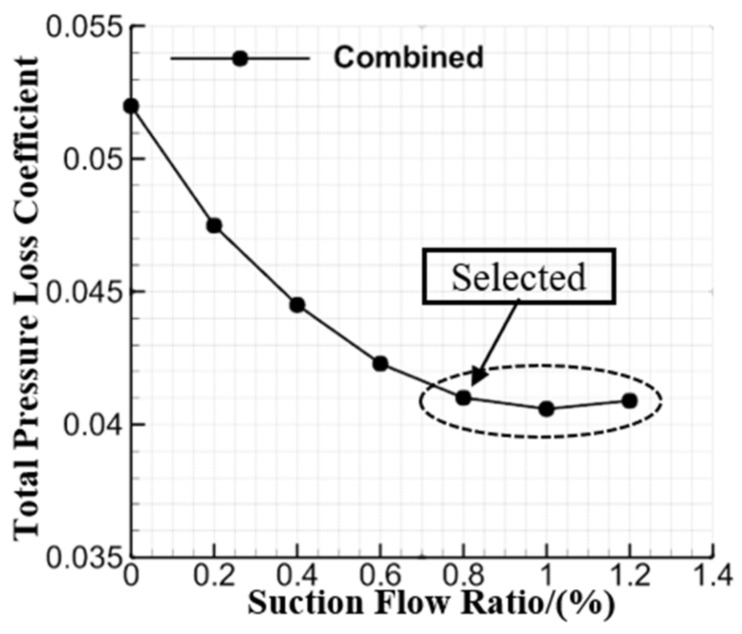
Loss of the combined configuration that varies with the suction flow ratio at the 0° incidence angle.

The EW suction slot meshes included 10,200 grid points, and the meshes of the suction slot inlet and EW surface were connected to each other with full non-matching method. For ensuring the suction flow ratio, this study applied a boundary condition with fixed mass-flow on the outlet boundary of the EW suction slot. Previous studies [33,34] have indicated that the EW BL suction has an optimal suction flow ratio. Figure 5 presents the loss, which varies as the suction flow ratio increases for the combined configuration, and the total pressure loss coefficient will be defined in Section 2.4. It can be found that the loss variation tends to be flat when the suction flow ratio exceeds 0.8%. Therefore, this study selected 0.8% as the suction flow ratio for the combined configuration, which will not consume excessive suction mass-flow. Besides this, to better evaluate the slotted and combined configurations for overall performance, the suction flow ratio of the combined configuration was fixed at 0.8% within the whole incidence angle range.

### 2.4. Numerical Method Validations

**Figure 6:**
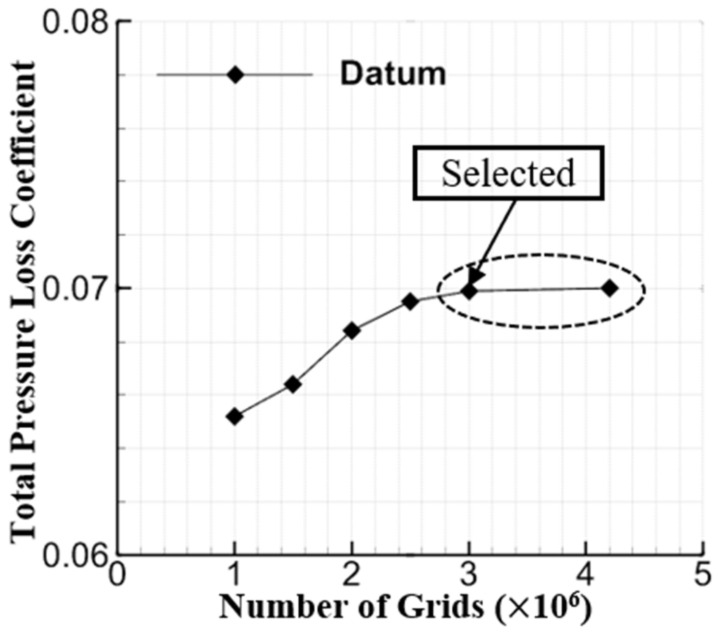
Grid independence validation for the datum cascade at the incidence angle of 0°.

The grid independence was first validated for verifying the simulation method feasibility. Figure 6 shows the total pressure loss coefficient of the datum cascade that varies as the grid number increases at the incidence angle of 0°, and Formula (1) presents the definition of the total pressure loss coefficient (w). The inlet total pressure and static pressure are represented by $P_{01}$ and $P_{1}$, respectively, and the local total pressure is represented by $P_{02}$. Considering the EW suction mass-flow of the combined configuration, Formula (2) defines its total pressure loss coefficient ($w_{s}$) based on [34,39,43]. $m_{1}$ is the inlet mass-flow of the cascade, and $m_{3}$ is the EW suction mass-flow. Besides this, $P_{03}$ presents the suction slot outlet total pressure, and $P_{01}$, $P_{02}$ and $P_{1}$ are consistent with the definitions in Formula (1). One can see from Figure 6 that the simulation results that converge behind its grid number exceed 3,000,000, eliminating the grid number’s impact on the simulation results.

(1) $$w=\frac{P_{01}-P_{02}}{P_{01}-P_{1}}$$

(2) $$w_{s}=\frac{\left(m_{1}-m_{3}\right)(P_{01}-P_{02})+m_{3}(P_{01}-P_{03})}{m_{1}\left(P_{01}-P_{1}\right)}$$

**Figure 7:**
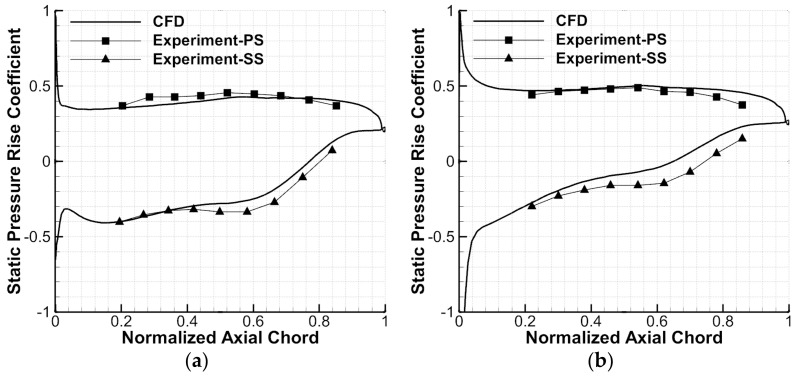
Blade surface static pressure rise coefficients comparison between simulations and experiments: (**a**) 0.5° incidence angle (**b**) 5° incidence angle.

Afterwards, the blade surface static pressure rise coefficients of the datum cascade were compared at 50%H for the simulations and experiments, which is shown in Figure 7. The experimental results have been repeatedly verified, with a high degree of reliability [33,38]. The static pressure rise coefficient ($C_{P}$) is defined in Formula (3), where the meanings of $P_{01}$ and $P_{1}$ are consistent with those in Formula (1), and $P_{2}$ represents the local static pressure. One can see from Figure 7 that a good agreement is shown between the computational and experimental results at the 0.5° incidence angle. At the 5° incidence angle, although there is a certain numerical deviation between the computational and experimental results on the blade SS, their trends are consistent and the computational results agree well with the experimental results on the blade PS. Overall, the reliability of the simulation method is preliminarily verified.

(3) $$C_{P}=\frac{P_{2}-P_{1}}{P_{01}-P_{1}}$$

**Figure 8:**
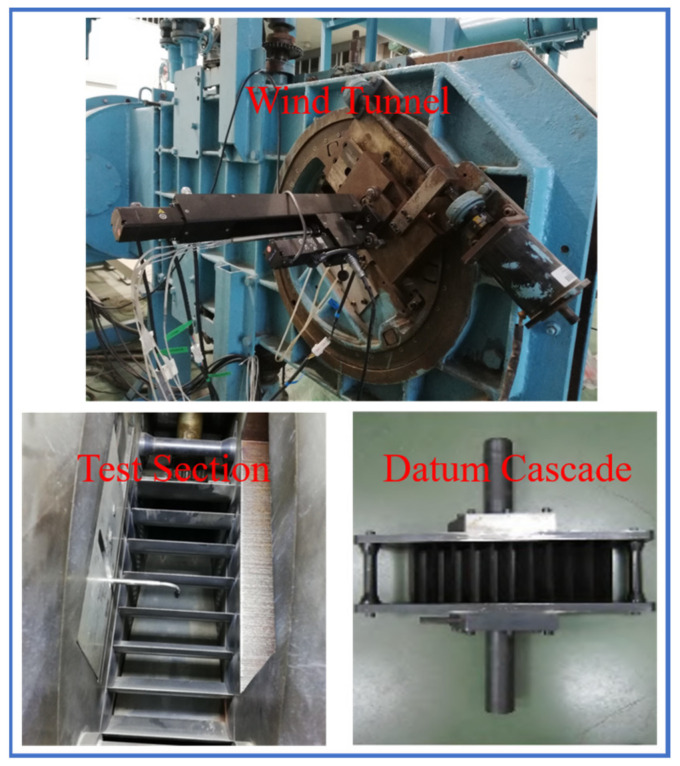
Some devices in the experiment.

**Figure 9:**
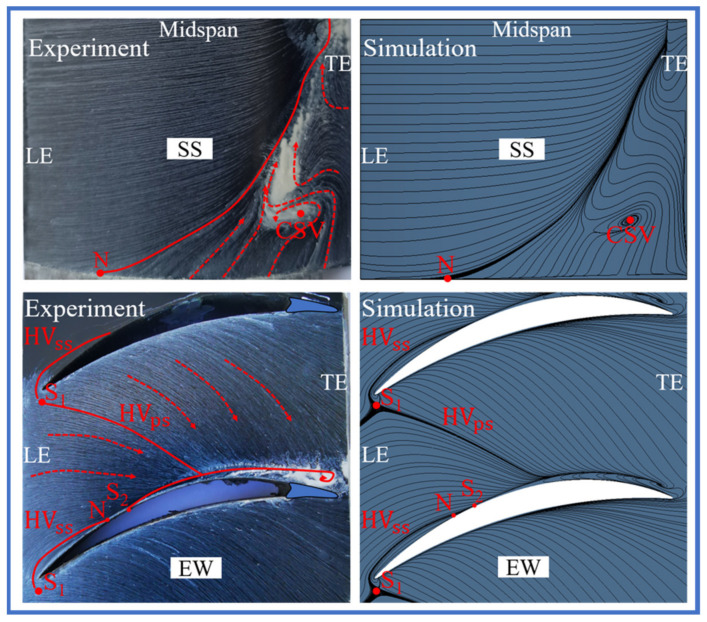
Simulation and experimental flow fields on the EW and blade SS of the datum cascade.

At last, the oil flow experiment was performed at the inlet condition with 12 mm BL thickness. Figure 8 presents the experimental devices for the datum cascade. The flow fields of the simulation and experiment are compared in Figure 9, and the computational settings were completely consistent with the experiment. Although the corner separation range gets larger at the inlet condition with 12 mm BL thickness compared with at the inlet condition with uniform flow, the simulation flow fields still show a great agreement with the experimental flow fields, including the corner separation range, concentrated shedding vortex (CSV), pressure and suction side leg of horseshoe vortexes (HV_PS_, HV_SS_) and positions of the critical points (saddle points S_1_, S_2_ and node point N). Therefore, it further proves the reliability of the simulation results at the inlet condition with uniform flow.

## 3. Performance Evaluation and Analysis

To evaluate the separation control effect of the slotted and combined configurations, the datum cascade, slotted configuration and combined configuration are compared for performance in detail from different aspects in this section: (1) At first, the flow patterns and aerodynamic parameters of the three cascades are compared in Section 3.1 and Section 3.2. (2) Subsequently, the vortical structures are selected for performance evaluation in Section 3.3. (3) Finally, the overall performance of the three cascades is presented and evaluated in Section 3.4. It is noted that since the flow structures of linear compressor cascades are symmetrical about the blade mid-span; this section performs the performance analysis in the blade height range of 0–50%H to highlight the details of the flow fields.

### 3.1. Velocity Contours and Flow Turning Angles

**Figure 10:**
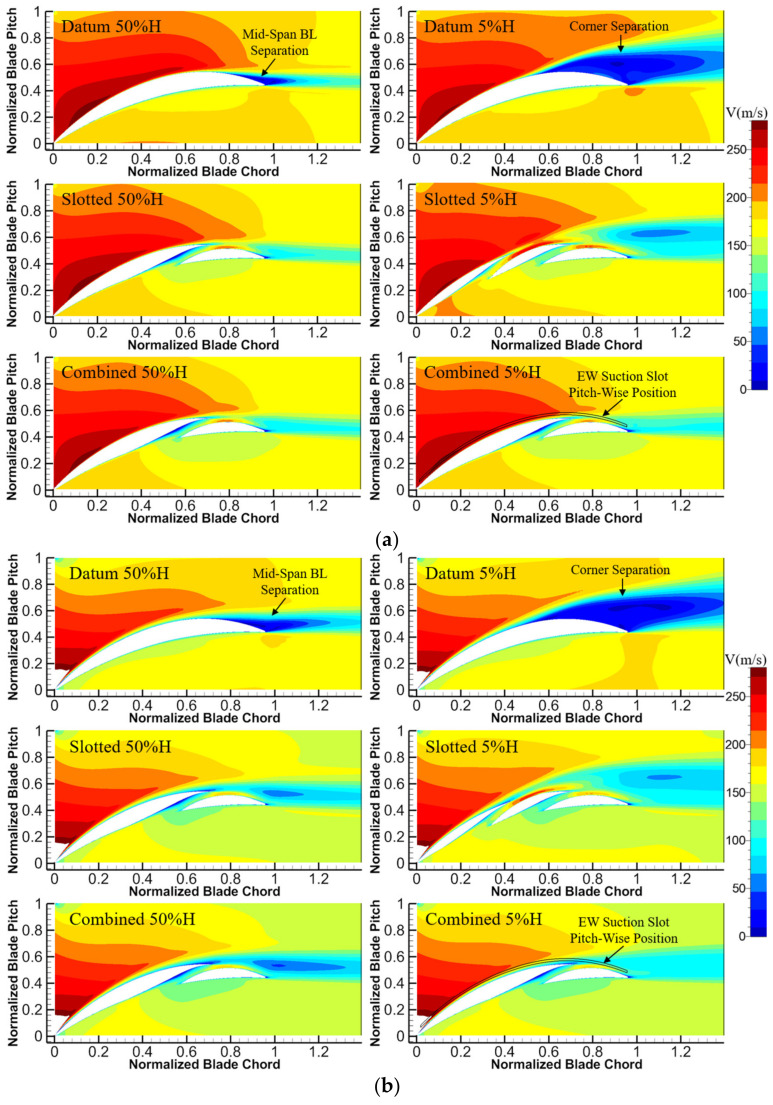
Velocity contours of the datum cascade, slotted configuration and combined configuration at different H and incidence angles: (**a**) 0° incidence angle (**b**) 6° incidence angle.

The velocity contours for the datum cascade, slotted configuration and combined configuration at different H and incidence angles are shown in Figure 10. One can see that whether at 50%H or 5%H, the slot structure can accelerate the fluid to be a high-momentum jet at the slot outlet under different incidence angles, whose velocity is basically consistent with the local main flow velocity of the blade SS side. It indicates the great self-adaptability of the slot structure designed in this study to incidence angles, which is beneficial for effectively blowing off the local low-momentum fluid, thus inhibiting the flow separation within the whole incidence angle range.

As a consequence, both the slotted and combined configurations can eliminate the mid-span BL separation for the datum cascade at the 0° and 6° incidence angles. However, the mixing effect of the whole-span slot jet and mainstream enhances the development of the wake loss, which causes the slotted and combined configurations to have basically the same wake loss developing range with the datum cascade at 50%H. At the 6° incidence angle, the combined configuration has a slightly larger wake loss than the slotted configuration at 50%H because of the stronger adverse pressure gradient.

The flow separation at 5%H of the datum cascade is also eliminated both in the slotted and combined configurations at the 0° and 6° incidence angles. The wake loss of the slotted and combined configurations is reduced compared with that of the datum cascade. However, the slotted configuration has slightly larger wake loss than the combined configuration, because the blade-end slot jet mixes with the mainstream and brings additional mixing loss.

In addition, under the same inlet velocity condition, the velocity distribution in the cascade main flow passage can basically reflect the pressure diffusion capacities of the three cascades. It can be found that the combined configuration experiences the smallest velocity at the axial position of blade TE, while the datum cascade experiences the largest. It indicates that the combined configuration has the largest pressure diffusion capacity, while the datum cascade has the smallest.

**Figure 11:**
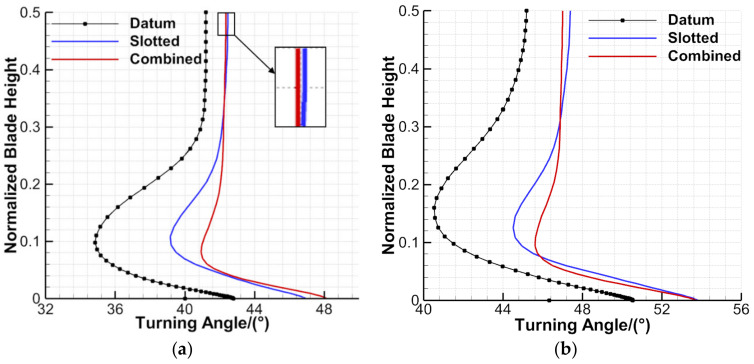
Flow turning angle distributions of the datum cascade, slotted configuration and combined configuration: (**a**) 0° incidence angle (**b**) 6° incidence angle.

Figure 11 presents the pitch-averaged flow turning-angle distributions of the datum cascade, slotted configuration and combined configuration along the blade span. One can see that at the 0° and 6° incidence angles, the slotted and combined configurations can increase the flow turning angles for the datum cascade within the full blade span. Near the blade midspan, the slotted comfiguration has larger flow turning angles than the combined configuration at the two incidence angles. Combined with the analysis of Figure 10, this is because the combined configuration has a stronger pressure diffusion capacity, and the main flow separates weakly at the blade TE under the larger adverse pressure gradient, leading to larger deviation angles (smaller flow turning angles). Besides this, in the range of 0–30%H, the combined configuration has fundamentally larger flow turning angles overall than the slotted configuration at the two incidence angles. This is because the combined configuration has a better effect than the slotted configuration in controlling the corner separation, leading to a further increment of the flow turning angle.

### 3.2. Total Pressure Loss and Limiting Streamlines

**Figure 12:**
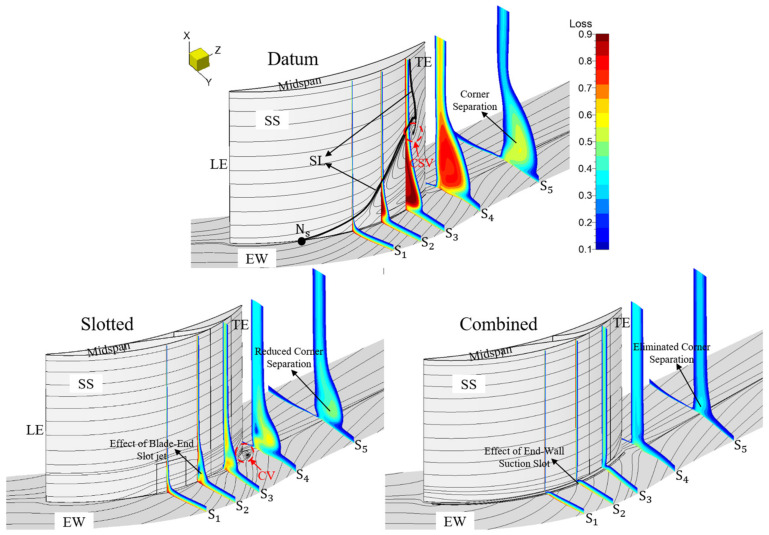
Total pressure loss and limiting streamlines for the datum cascade, slotted configuration and combined configuration at the 0° incidence angle.

Figure 12 presents the passage total pressure loss and limiting streamlines of the datum cascade, slotted configuration and combined configuration at the 0° incidence angle, and the axial positions of the five planes of S_1_, S_2_, S_3_, S_4_ and S_5_ perpendicular to the Z axis are, respectively, 50%Ca, 67%Ca, 83%Ca, 105%Ca and 150%Ca. At the 0° incidence angle, a relatively serious corner separation exists in the datum cascade with the occurrence of the mid-span BL separation. The corner SL starting position is about 30%Ca away from the blade LE, and the corner separation occupies 30% of the blade span. The corner separation leads to certain passage blockage and total pressure loss, thus weakening the pressure diffusion capacity of the datum cascade. In addition, the CSV exists in the datum cascade passage.

In the slotted configuration, the low-momentum fluid in the BL can be effectively re-energized by the slot high-momentum jet. Hence, the whole-span slot jet removes the mid-span BL separation while the blade-end slot jet effectively restricts the EW secondary flow migration. Under the coupled action of the high-momentum jets generated by the whole-span and blade-end slots, the elimination of the CSV and the reduction in the corner separation are achieved. However, the corner vortex (CV) occurs in the corner between the EW and blade SS since the adverse pressure gradient gets stronger.

In the combined configuration, the whole-span slot jet removes the mid-span BL separation, too. Since the low-momentum fluid concentrated in the corner between the EW and blade SS can be effectively sucked off by the EW suction slot, the formation of the EW secondary flow is inhibited. Observing the blade SS limiting streamlines near the EW and the total pressure loss on the S_2_ plane, one can see that the EW suction slot behaves better than the blade-end slot in inhibiting the EW secondary flow migration. Additionally, the mixing loss between the mainstream and the blade-end slot jet is avoided, hence the loss caused by the corner separation can be further reduced. Meanwhile, the generation of CV is suppressed by the whole-chord-length EW suction slot, contributing to a further reduction in the corner separation. Therefore, the combined configuration basically eliminates the corner separation.

**Figure 13:**
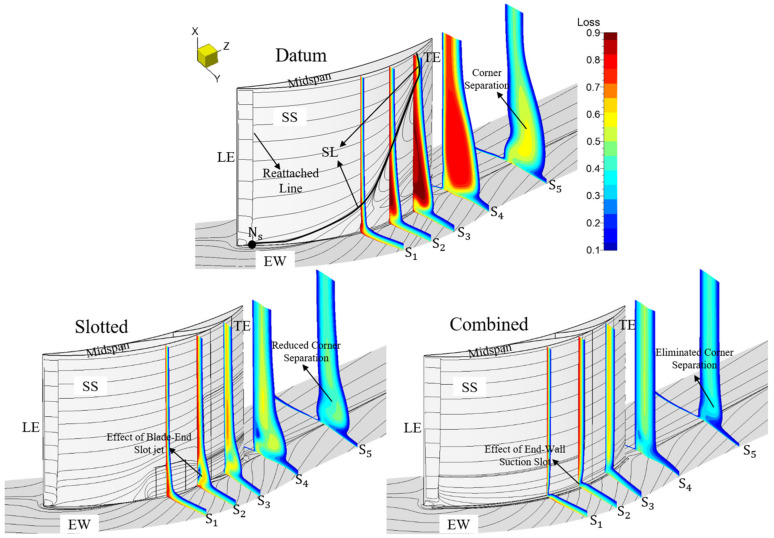
Total pressure loss and limiting streamlines for the datum cascade, slotted configuration and combined configuration at the 6° incidence angle.

Figure 13 presents the passage total pressure loss and limiting streamlines of the datum cascade, slotted configuration and combined configuration at the 6° incidence angle. One can see that as the incidence angle increases, the corner separation range becomes larger in the datum cascade while the TE separation also gets more serious. The corner SL starting position moves toward the blade LE, and the corner separation occupies about 40% of the blade span. Additionally, due to the larger incidence angle, the incoming flow is separated directly at the blade LE. Afterwards, a reattached line is formed because of the reattachment of the separated flow under the effect of the main stream.

However, the serious flow separation in the datum cascade still can be controlled by the slotted and combined configurations. Consistent with the circumstances at the 0° incidence angle, the mid-span BL separation is removed both in the slotted and combined configurations. The corner separation is reduced in the slotted configuration, while it can be basically eliminated in the combined configuration. As a whole, whether under small or large incidence angles, the slotted and combined configurations have a good effect for inhibiting the EW secondary flow migration. However, the EW suction slot behaves better than the blade-end slot in controlling the EW secondary flow migration, and the mixing loss of the mainstream with the blade-end slot jet is also avoided in the combined configuration. Consequently, the combined configuration has smaller total pressure loss in the corner than the slotted configuration.

### 3.3. Vortical Structures

**Figure 14:**
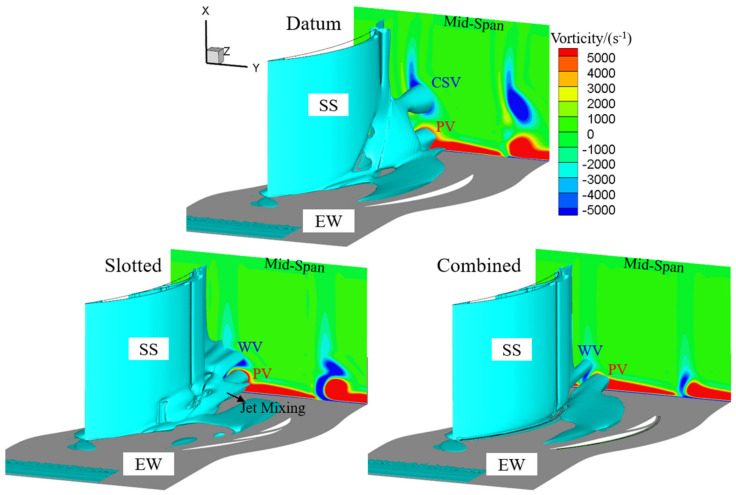
3D corner separation structures extracted by Q = 1,000,000 s^−2^ iso-surface and contoured by streamwise vorticity at the 0° incidence angle.

The vortical structures of the datum cascade, slotted configuration and combined configuration at the 0° incidence angle are compared in Figure 14, where the 3D corner separation structures are extracted by Q = 1,000,000 s^−2^ iso-surface and the plane (150%Ca) perpendicular to z axis is contoured by streamwise vorticity. One can see that two vortical structures of passage vortex (PV) and CSV mainly exist in the datum cascade passage, contributing to the serious loss in the corner.

In the slotted configuration, because the high-momentum slot jet effectively re-energizes the low-momentum BL on the EW and blade SS, the PV is slightly reduced and the CSV is removed. However, the flow turning angle and cascade effective outlet area increase due to the reduction in the corner separation and the elimination of the mid-span BL separation, thus leading to a stronger pressure diffusion capacity. Under the enhanced adverse pressure gradient, another vortex structure named a ‘wall vortex’ (WV) occurs. According to the previous study [17], the WV is formed by the mutual interference and separation of the slot jet, main stream and EW secondary flow. However, the new generated WV has lower strength than the CSV. Consequently, the vortex structures of the datum cascade are improved by the slotted configuration.

In the combined configuration, the CSV is also removed. Because the EW BL suction slot has a better effect in suppressing the EW secondary flow development than the blade-end slot, the mutual interference of the slot jet, main stream and EW secondary flow is reduced in the combined configuration. Therefore, the combined configuration has a further reduced WV than the slotted configuration. In addition, as a result of the suction effect of the BL suction slot on the EW BL, the PV in the combined configuration is also weaker than that of the slotted configuration.

**Figure 15:**
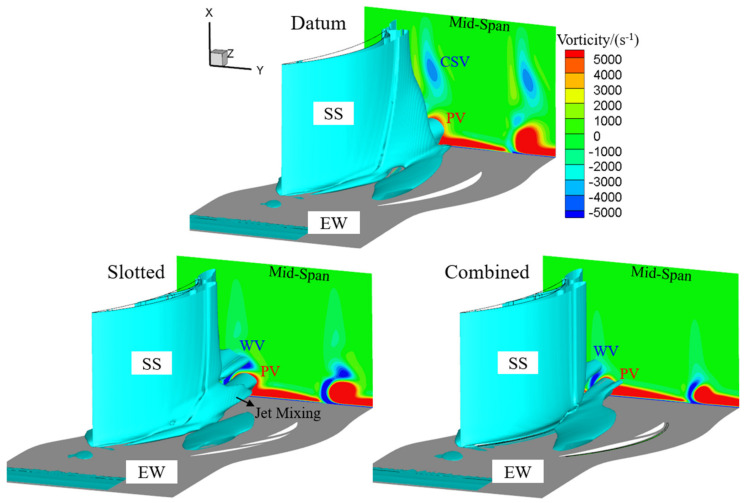
3D corner separation structures extracted by Q = 1,000,000 s^−2^ iso-surface and contoured by streamwise vorticity at the 6° incidence angle.

Figure 15 presents the vortical structures of the datum cascade, slotted configuration and combined configuration at the 6° incidence angle. One can see that the CSV gets weaker and the PV gets stronger in the datum cascade passage as the incidence angle gets larger. In the slotted configuration, the flow separation still can be effectively controlled under the coupled blowing effect of the high-momentum jets generated by the whole-span and blade-end slots, bringing the elimination of the CSV and the reduction in the PV. However, as the adverse pressure gradient is enhanced, the WV is also formed due to the interference and separation of the slot jet, main stream and EW secondary flow.

The combined configuration still can better control the corner separation than the slotted configuration at the 6° incidence angle. Compared with those in the slotted configuration, the WV and PV are further reduced in the combined configuration. By contrast, as a whole, the combined configuration shows a better effect in improving the vortex structures for the datum cascade than the slotted configuration.

### 3.4. Overall Performance Comparison

**Figure 16:**
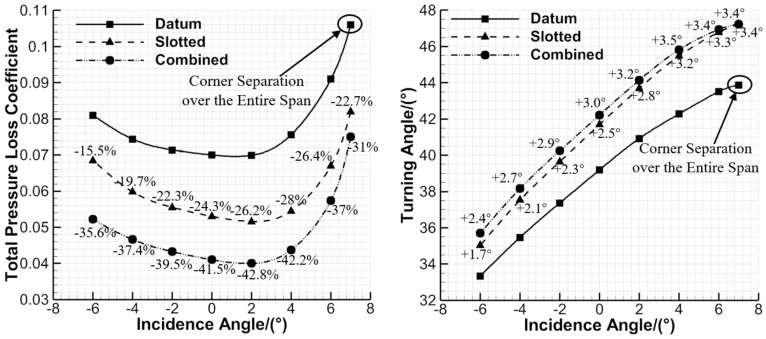
Aerodynamic parameters of the datum cascade, slotted configuration and combined configuration that vary with incidence angles.

To compare the datum cascade, slotted configuration and combined configuration for overall performance, Figure 16 presents their aerodynamic parameters (total pressure loss coefficient and flow turning angle) that vary with incidence angles. It is evident that the total pressure loss of the datum cascade can be reduced within −6°~7° incidence angles by the slotted and combined configurations. This is advantageous to broadening the available incidence angle range of compressor blades, thus increasing the stability margin of compressors. According to the analysis in Section 3.2, the combined configuration behaves better than the slotted configuration in controlling the EW secondary flow development, while the mixing loss caused by the main flow and blade-end slot jet is avoided. Therefore, the combined configuration has smaller total pressure loss within −6°~7° incidence angles.

The total pressure loss of the datum cascade can be reduced by 23.2% on average in the slotted configuration, while it can be reduced by 38.4% on average in the combined configuration. In addition, the combined configuration has an average reduction effect to the total pressure loss of the datum cascade from −6° to 7°, while the slotted configuration has a basically enhanced reduction effect. This is because the corner separation gradually becomes more serious as the incidence angle increases, and the separation loss reduced by the blade-end slot jet is gradually greater than the mixing loss with the main flow.

The slotted and combined configurations both increase the flow turning angles for the datum cascade within −6°~7° incidence angles. The combined configuration has larger flow turning angles than the slotted configuration, which further confirms the analysis of Figure 11. Compared with that of the datum cascade, the flow turning angle can be increased by 2.7° on average in the slotted configuration, while it can be increased by 3.1° on average in the combined configuration. Therefore, by comparison as a whole, the combined configuration behaves better than the slotted configuration in controlling the corner separation and mid-span BL separation.

## 4. Effect of Blade Solidity

**Figure 17:**
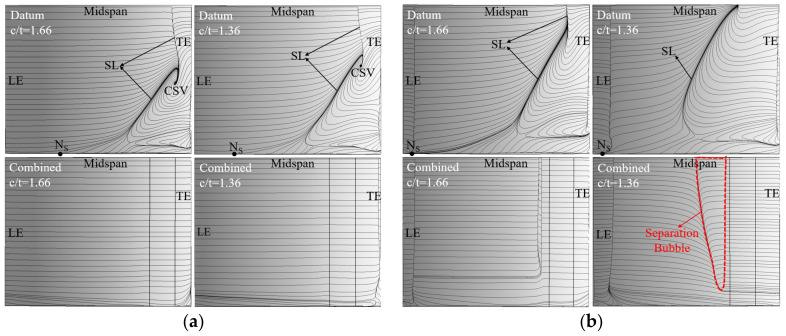
Blade SS limiting streamlines of the datum cascade and combined configuration at c/t = 1.66 and 1.36: (**a**) 0° incidence angle (**b**) 6° incidence angle.

To study the impact of c/t on the separation control effect of the combined configuration, the c/t is decreased from 1.66 to 1.36 by increasing the value of t. However, the SL will move toward the blade LE with the decrease in c/t, causing a movement of suitable slot outlet position [29,30]. Hence, the whole-span slot outlet was located at 70%Ca in the combined configuration for the c/t of 1.36. Figure 17 presents the blade SS limiting streamlines of the datum cascade and combined configuration at c/t = 1.66 and 1.36. It can be found that the corner separation range of the datum cascade gets larger as the c/t decreases from 1.66 to 1.36. This is because the lower c/t leads to a higher pitch-wise pressure gradient, which transports more low-momentum fluid into the corner between the EW and blade SS, thus enhancing the corner separation.

Although the main flow separates prior to the whole-span slot at the 6° incidence angle when the c/t = 1.36, the separated flow reattaches to the blade SS to form a separation bubble because of the whole-span slot jet blowing effect. As a whole, the mid-span BL separation and corner separation of the datum cascade can be both basically removed in the combined configuration at c/t = 1.66 and 1.36. It is because that the EW suction slot effectively sucks off the low-momentum fluid concentrated in the corner, which restricts the EW secondary flow formation. Meanwhile, the whole-span slot jet blows off the local low-momentum fluid on the blade SS, thus eliminating the mid-span BL separation.

**Figure 18:**
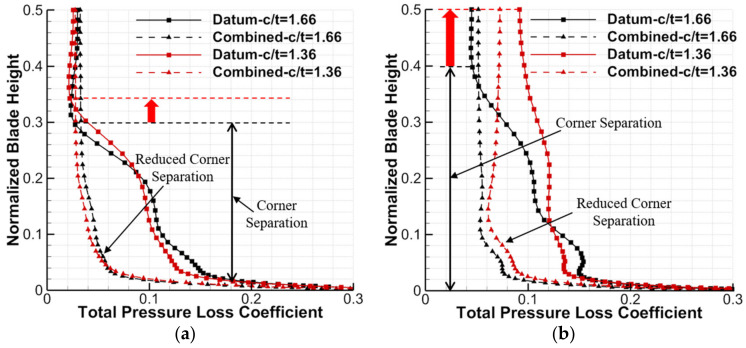
Total pressure loss distributions for the datum cascade and combined configuration at c/t = 1.66 and 1.36: (**a**) 0° incidence angle (**b**) 6° incidence angle.

Figure 18 shows the pitch-averaged total pressure loss distributions for the datum cascade and combined configuration at c/t = 1.66 and 1.36, which can intuitively reflect the loss level and the spanwise range of the corner separation. One can see that as the c/t decreases from 1.66 to 1.36, the corner separation spanwise range of the datum cascade expands from 30%H to 34%H at the 0° incidence angle, while it expands from 40%H to 50%H at the 6° incidence angle. However, consistent with the analysis of Figure 17, the combined configuration can basically eliminate the corner separation at c/t = 1.66 or 1.36, which contributes to a great reduction in the total pressure loss. However, although the combined configuration removes the mid-span BL separation for the datum cascade, the total pressure loss near the midspan of the combined configuration is slightly larger than that of the datum cascade at the condition with relatively weak mid-span BL separation. According to [28,30], this is because the mixing effect of the whole-span slot jet and main stream enhances the wake loss developing range.

**Figure 19:**
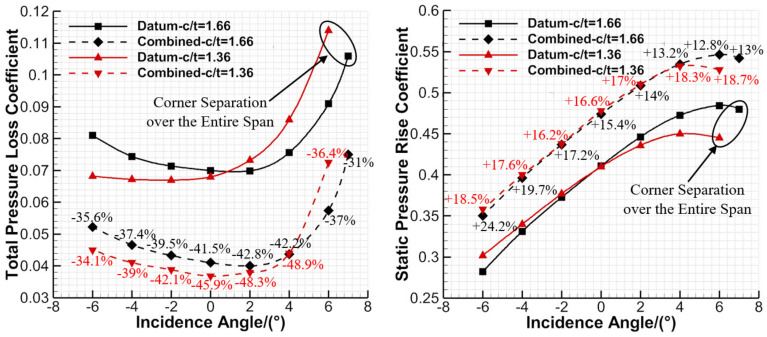
Aerodynamic parameters of the datum cascade and combined configuration that vary with incidence angles at c/t = 1.66 and 1.36.

Figure 19 presents the aerodynamic parameters of the datum cascade and combined configuration that vary with incidence angles at c/t = 1.66 and 1.36. One can see that the datum cascade experiences higher loss and smaller static pressure rise coefficient at the positive incidence angles as the c/t decreases from 1.66 to 1.36, which is consistent with the study of Sans et al. [44]. This is because lower c/t brings higher blade loading, thus narrowing the cascade available incidence angle range. Because of the combined control effect of the EW BL suction and whole-span slot jet, the combined configuration still can significantly reduce the total pressure loss and improve the static pressure rise coefficient.

For the combined configuration with c/t = 1.66 and 1.36, the total pressure loss is reduced by 38.4% and 42.1%, respectively, on average, while the averaged static pressure rise coefficient is increased by 16.2% and 17.6%, respectively. One can see that the combined configuration shows a stronger inhibiting effect on the flow separation as the c/t decreases, meaning it has a good adaptability to lower c/t conditions or higher blade loading conditions. Additionally, the combined configuration with c/t = 1.36 can achieve stronger pressure diffusion capacity than the datum cascade with c/t = 1.66, while its total pressure loss is also smaller. Therefore, the combined configuration is advantageous to the improvement of the aero-engine thrust-to-weight ratio through decreasing the single-stage blade number of compressors.

## 5. Conclusions

To investigate a better control strategy for corner separation and mid-span BL separation, two new flow control configurations are proposed in this study. One is the slotted configuration composed of blade-end and whole-span slots, and the other is the combined configuration with EW suction slot and whole-span slot. Based on experimentally validated simulations, the datum cascade, slotted configuration and combined configuration are evaluated in detail for performance within the incidence angle range of −6°~7°. Besides this, the effect of c/t on the cascade performance is investigated. Conclusions are drawn as follows:

(1) Under the re-energization of the high-momentum slot jets to the local low-momentum fluid, the slotted configuration reduces the corner separation for the datum cascade while eliminating the mid-span BL separation. The EW secondary flow development can be effectively controlled by the blade-end slot jet, but it brings additional mixing loss with the main flow. Additionally, the slotted configuration eliminates the CSV and reduces the PV, but the new generated WV will lead to additional loss.

(2) The low-momentum fluid concentrated in the corner can be effectively sucked off by the EW suction slot, which inhibits the formation of the EW secondary flow. Therefore, the combined configuration eliminates the CSV and further reduces the PV and WV. Owing to the combination of the EW BL suction and whole-span slot jet, the combined configuration basically removes the corner separation and mid-span BL separation for the datum cascade.

(3) In the whole incidence angle range, the total pressure loss and flow turning angle of the datum cascade can be increased by −23.2% and 2.7°, respectively, on average in the slotted configuration, while they can be increased by −38.4% and 3.1°, respectively, on average in the combined configuration. Consequently, the combined configuration is more beneficial than the slotted configuration in improving the stability margin and pressure diffusion capacity for compressor blades.

(4) The decrease in c/t leads to a higher pitch-wise pressure gradient, which transports more low-momentum fluid into the corner between the EW and blade SS, thus enhancing the corner separation of the datum cascade. However, the combined configuration has a good adaptability to lower c/t conditions. In the combined configuration with c/t of 1.66 and 1.36, the total pressure loss is reduced by 38.4% and 42.1%, respectively, on average, while the averaged static pressure rise coefficient is increased by 16.2% and 17.6%, respectively.

(5) The combined configuration with lower c/t can achieve a stronger pressure diffusion capacity and smaller loss than the datum cascade with higher c/t. As a result, it is beneficial for achieving a higher aero-engine thrust-to-weight ratio by applying the combined configuration in compressors to decrease the compressor single-stage blade number.

## Figures and Tables

**Table 1:** Datum cascade, geometric and aerodynamic parameters.

Parameters	Values
Blade height H/mm	100
Chord c/mm Blade pitch t/mm	63 37.95
Aspect ratio H/c	1.59
Blade solidity c/t	1.66
Geometric inlet angle $\beta_{1k}$/(°)	40.17
Geometric outlet angle $\beta_{2k}$/(°) Stagger angle $\beta_{s}$/(°)	−13.21 15.4
Inlet Mach number $Ma_{1}$	0.7
Reynolds number $Re_{C}$	$7.7\times 10^{5}$

## Nomenclature

$\beta_{1}$	Inlet flow angle
$\beta_{1k}$	Geometric inlet angle
$\beta_{2}$	Outlet flow angle
$\beta_{2k}$	Geometric outlet angle
$\beta_{s}$	Stagger angle
w	Total pressure loss coefficient without suction mass-flow
$w_{s}$	Total pressure loss coefficient with suction mass-flow
Ca	Axial blade chord
c	Blade chord
c/t	Blade solidity
$C_{P}$	Static pressure rise coefficient
H	Blade height
H/c	Aspect ratio
$Ma_{1}$	Inlet Mach number
$m_{1}$	Inlet mass-flow
$m_{3}$	End-wall suction mass-flow
$P_{1}$	Inlet static pressure
$P_{01}$	Inlet total pressure
$P_{2}$	Local static pressure
$P_{02}$	Local total pressure
$P_{03}$	Total pressure at end-wall suction slot outlet
$Re_{C}$	Blade-chord-based Reynolds number
$R_{1}$	Outlet curve radius of blade-end slot lower wall
$R_{2}$	Outlet curve radius of whole-span slot lower wall
t	Blade pitch
$X_{1}$	Outlet throat width of blade-end slot
$X_{2}$	Outlet throat width of whole-span slot
$y^{+}$	Dimensionless wall first layer grid size
**Abbreviations**	
AO_1_	Axial overlap of the blade-end slot
AO_2_	Axial overlap of the whole-span slot
BL	Boundary layer
CV	Corner vortex
CSV	Concentrated shedding vortex
EW	End-wall
${\mathrm{HV}}_{\mathrm{PS}}$	Horseshoe vortex pressure side leg
${\mathrm{HV}}_{\mathrm{SS}}$	Horseshoe vortex suction side leg
LE	Leading edge
N	Node
PS	Pressure surface
PV	Passage vortex
RANS	Reynolds averaged Navier–Stokes
SL	Separation Line
SS	Suction surface
SA	Spalart–Allmaras
S	Saddle point
TE	Trailing edge
WV	Wall vortex
